# Complete genomic sequence of the *Vibrio alginolyticus* bacteriophage Vp670 and characterization of the lysis-related genes, *cwlQ* and *holA*

**DOI:** 10.1186/s12864-018-5131-x

**Published:** 2018-10-11

**Authors:** Peng Luo, Long Yun, Yingying Li, Yushun Tian, Qiuting Liu, Wen Huang, Chaoqun Hu

**Affiliations:** 1 CAS Key Laboratory of Tropical Marine Bio-resources and Ecology (LMB), Guangdong Provincial Key Laboratory of Applied Marine Biology (LAMB), South China Sea Institute of Oceanology, Chinese Academy of Sciences, Guangzhou, 501301 People’s Republic of China; 2 South China Sea Bio-Resource Exploitation and Utilization Collaborative Innovation Center, Guangzhou, 510301 People’s Republic of China; 3 University of Chinese Academy of Sciences, Beijing, 100049 China

**Keywords:** *Vibrio alginolyticus*, Bacteriophage, Holin, Endolysin

## Abstract

**Background:**

Biocontrol of bacterial pathogens by bacteriophages (phages) represents a promising strategy. *Vibrio alginolyticus*, a gram-negative bacterium, is a notorious pathogen responsible for the loss of economically important farmed marine animals. To date, few *V. alginolyticus* phages have been successfully isolated, and only three complete genome sequences of them have been released. The limited available phage resources and poor genomic data hamper research on *V. alginolyticus* phages and their applications for the biocontrol of *V. alginolyticus*.

**Results:**

We isolated a phage, Vp670, against the *V. alginolyticus* strain E06333 and obtained its full genomic sequence. It contains 43,121 nucleotides with a GC content of 43.4%, and codes for 49 predicted open reading frames. Observation by electron microscope combined with phylogenetic analysis of DNA polymerase indicates that Vp670 belongs to the subfamily Autographivirinae in the family Podoviridae. *orf3* (designated *holA*) and *orf8* (designated *cwlQ*) are predicted to encode a holin (HolA) and an endolysin (CwlQ), respectively. Expression of *holA* alone or coexpression of *holA* and *cwlQ* from within arrested the growth of *Escherichia coli* and *V. alginolyticus* while the expression of *cwlQ* alone had no effect on the growth of them. Further observation by transmission electron microscopy revealed that the expression of *holA* vanished the outer membrane and caused the release of cellular contents of *V. alginolyticus* and the coexpression of *holA* and *cwlQ* directly burst the cells and caused a more drastic release of cellular contents. Expression of *cwlQ* alone in *V. alginolyticus* did not cause cytomorphological changes.

**Conclusions:**

Phage Vp670 is a *V. alginolyticus* phage belonging to the family of Podoviridae. The genome of Vp670 contains a two-component lysis module, which is comprised of *holA* and *cwlQ*. *holA* is predicted to encode for the holin protein, HolA, and *cwlQ* is predicted to encode for the endolysin protein, CwlQ. Both *holA* and *cwlQ* likely play important roles during the release of phage progeny.

**Electronic supplementary material:**

The online version of this article (10.1186/s12864-018-5131-x) contains supplementary material, which is available to authorized users.

## Background

*Vibrio alginolyticus*, a gram-negative bacterium, is an opportunistic pathogen to marine animals and human beings. It has been reported to be an important pathogen responsible for economic losses in several aquatic species [1]. Traditionally, *V. alginolyticus* and other pathogenic *Vibrio* species are controlled using antibiotics; however, this method is becoming less and less effective due to the emergence of antibiotic-resistant strains, and environmental and ecological problems related to widespread antibiotic use [1, 2]. It is estimated that most bacteria (90%) isolated from marine environments are resistant to more than one antibiotic and that 20% of them are resistant to at least five antibiotics [3, 4]. Antibiotics abuse is known to accelerate the emergence of multi-drug-resistant bacteria. Therefore, it is urgent to explore new ways to prevent infection by pathogenic *V. alginolyticus* and other *Vibrio* species*.*

Bacteriophages (phages) belong to a category of viruses that infect and lyse bacteria and are natural and abundant in the marine environment. Phages play important roles in, for example, structuring bacterial diversity and succession in the ocean, promoting biogeochemical cycling of elements, and driving horizontal gene transfer [5, 6]. In recent years, phages have gained much attention as they have been found to have some advantages over antibiotics in terms of controlling bacterial infection [7–9]. Antibiotics persist in the environment while phages proliferate only when a specific bacterial host is present. Therefore, unlike antibiotics, phages do not disturb the normal flora in animals and humans [7–9] and theoretically do not cause the diffusion of drug-resistance genes. Endolysins, lytic enzymes of bacteriophages, are proteins synthesized at the end of the lytic cycle, and they destroy the cell wall peptidoglycan (PG) to facilitate the release of viral progeny [10]. Both phages and phage endolysins are alternative ways to prevent pathogens, especially those with multi-drug resistance. Using purified endolysins may have certain advantages over whole phages [11, 12]. One significant advantage of endolysins is that, unlike antibiotics and whole phages there have been no reports of the development of bacterial resistance [11, 12].

To date, few *V. alginolyticus* phages have been successfully isolated [1, 13, 14], and only three complete genome sequences of their phages have been released [13]. The limited available phage resources and poor genomic data hamper research on *V. alginolyticus* phages and their use in pathogen biocontrol. In the present study, we sequenced the *V. alginolyticus* phage Vp670 that was isolated from marine water (Shenzhen, China) and performed bioinformatic analysis. The functions of the lysis-related genes, *cwlQ* and *holA*, were examined by the expression of each gene or fused genes in *Escherichia coli* and the host of the phage, *V. alginolyticus*. This study will, to some degree, create a path to the biocontrol of *V. alginolyticus*.

## Methods

### Bacterial strains and growth conditions

*V. alginolyticus* E06333 was isolated from the ulcer of a diseased and cage-cultured fish (*Epinephelus daemelii*) in Zhanjiang (Guangdong Province, China) in 2006. The ulcer of the fish was externally disinfected with 75% ethanol and was sampled with a cotton swab. Then the fish were provisionally cultured with a tank with seawater until it died. The animal experiment and sample collection were performed in accordance with the guidelines according to the Department of Scientific Research and Planning of South China Sea Institute of Oceanology, Chinese Academy of Sciences. Strain E06333 is resistance to ampicillin and sulfamethoxazole. *V. alginolyticus* E06333, *E. coli* DH5α and their derivative strains (Additional file 1: Table S1) were cultured in Luria-Bertani (LB) broth or on an LB agar plate at 30 °C.

### Isolation and purification of phage Vp670

The isolation and purification of phage Vp670 was performed as described previously, with minor modifications [15]. *V. alginolyticus* E06333 was cultured overnight as an indicator host for a plaque assay. Sewage water samples obtained from Dayawan, Yangjiang, and Zhanjiang in Guangdong, China were centrifuged at 10,000 g for 10 min, and then the supernatants were filtered using a sterile 0.45-μm filter. The filtrates were mixed with the same volume of 2 × LB broth and then were added with 1% overnight culture of E06333 cells followed by incubation at 30 °C for 12 h. The lytic culture samples (which remained transparent) were centrifuged, the supernatant was pipetted out, and was then filtered through a sterile 0.22-μm filter. The filtrate was used to isolate phages through the double-plate method [15]. A single plaque was picked out of the plates and diluted in SM buffer composed of 0.05 M Tris-HCl (pH 7.5), 0.1 M NaCl, 10 mM MgSO_4,_ and 1% gelatin for subculture and purification.

### Morphological observation by transmission electron microscopy

The morphology of phage Vp670 and the bacterial cells were observed with a Hitachi transmission electron microscope (TEM) after they had been negatively stained with 2% (*W*/*V*) phosphotungstic acid according to the method previously described [16].

### The replication parameters of phage Vp670

One-step growth curve for phage Vp670 was analyzed as previously described [17, 18] to determine the replication characteristics of the phage. In brief, an overnight-grown culture of strain E06333 at 30 °C was inoculated into fresh LB at a ratio of 1% and grown until the optical density (OD, 600 nm) reached at 1.0 (~ 3 × 10^8^ CFU/ml). The bacterial culture was 10-fold diluted to ~ 3 × 10^7^ CFU/ml and then 500-μl diluted culture was mixed with the same volume of phage Vp670 solution (Multiplicity of Infection, MOI = 1). The bacteria/phage mixture (1 ml) was added to 19 ml of LB broth. 100-μl aliquots from each sample were collected every 10 min for the first 60 min, and at 90 min, 120 min, 180 min, and 240 min. The samples were immediately diluted as needed and then incubated on double-layer plates to obtain phage plaques and determine the phage titers. The burst size (Bs) was calculated as Bs = Pt/P0, where Pt is the maximum phage titer at the plateau phase and P0 is the initial infective titer [17, 18]. The experiments contained three replicates.

### Adsorption rate of phage Vp670

The phage adsorption rate assay was performed as previously described, with some minor modifications [19]. The host E06333 cells were cultured overnight. LB broth and 1% overnight-grown *V. alginolyticus* were incubated at 30 °C until the optical density (OD, at 600 nm) of this culture reached 1.0. Five hundred microliters of the culture (OD = 1.0) was mixed with same volume of phage Vp670 solution (MOI = 0.1) and then suspended into 19-ml LB broth. This point was regarded as the start time point for sampling. A 1-ml sample was taken every 2 min until 10 min and immediately centrifuged at 8000 rpm for 2 min to separate the bacteria from the suspension composed of free phage. The supernatant was discarded, and then the pellet was resuspended, properly diluted and plated for the phage titer.

### Phage genome extraction and genomic sequencing

After centrifugation of the phage lysate at 10,000×g for 10 min, the supernatant was filtered through a 0.45-μm filter. Then, the filtrate was used for genome extraction using the Phage Genome Extraction Kit (KnoGen, China) according to the manufacturer’s instructions. The genomic DNA was sequenced by Suzhou GENEWIZ Biotechnology Co., LTD using an Illumina Miseq to obtain the complete genome sequence of phage Vp670.

### Bioinformatic analysis of phage genome

Gene prediction and functional annotation were conducted using the RAST server (http://rast.nmpdr.org/) followed by correction with BLAST searches. SMART online (http://smart.embl-heidelberg.de/ /) was used to predict putative conserved domains. Signal peptides and transmembrane domains were predicted using SignalP 4.0 Server (http://www.cbs.dtu.dk/services/SignalP/) and TMHMM v2.0 Server (http://www.cbs.dtu.dk/services/TMHMM/), respectively. A hydrophilicity plot of predicted proteins was produced using the ProtScale (https://web.expasy.org/protscale/). To determine the taxonomic status of bacteriophage Vp670, a phylogenetic tree was constructed based on DNA polymerase (DNAP) [20]. The sequences of DNAP from different members of the family Podoviridae were retrieved from GenBank. The sequences were aligned for the construction of a phylogenetic tree using MEGA 6.0 based on the neighbor-joining method. The GenBank accession numbers of these phages are listed in (Additional file 2: Table S2).

### Cloning and expression of *holA*, *cwlQ*, and *holA*-*cwlQ* in *Escherichia coli* and *V*. *alginolyticus*

The complete *holA*, *cwlQ,* and *holA*-*cwlQ* genes were amplified using PrimSTAR Max DNA Polymerase (Takara, China). The *holA*-*cwlQ* gene fusion was constructed by overlap extension PCR. To avoid digestion steps during the expression vector construction, the vector fragment was amplified using pBAD18-Kan as template and then recombinated with the previously amplified *holA*, *cwlQ,* and *holA*-*cwlQ* genes, respectively. The resulting plasmids were transformed into competent *E. coli* DH5α cells (TaKaRa, China) according to the manufacturer’s instructions. The pBAD18-*holA*, pBAD18-*cwlQ*, and pBAD18-*holA*-*cwlQ* plasmids were transferred into *V. alginolyticus* by electroporation according to a previously published method [21]. All the primers used in this study were listed in (Additional file 3: Table S3).

The bacterial cells containing the above-mentioned plasmids were cultured (with kanamycin [50 μg/ml] and 0.3% of D-glucose) until the OD_600nm_ reached at about 0.6. The cells were centrifuged and resuspended with fresh LB medium. The concentration of the resuspended cells were adjusted to OD_600nm_ = 0.5, and then were diluted by 10^6^. 0.1 ml of each diluted culture was spread on an LB agar plate containing kanamycin and L-arabinose (0.2%). Three independent replicates of the experiment were performed. Each replicate sample had three independent dilutions and numbers of clones on the plates. In addition, the resuspended cultures of *V. alginolyticu*s were supplemented with kanamycin and L-arabinose and incubated for 30 min at 30 °C and negatively stained followed by observation using TEM.

## Results

### Bacteriophage isolation and morphology

**Fig. 1:**
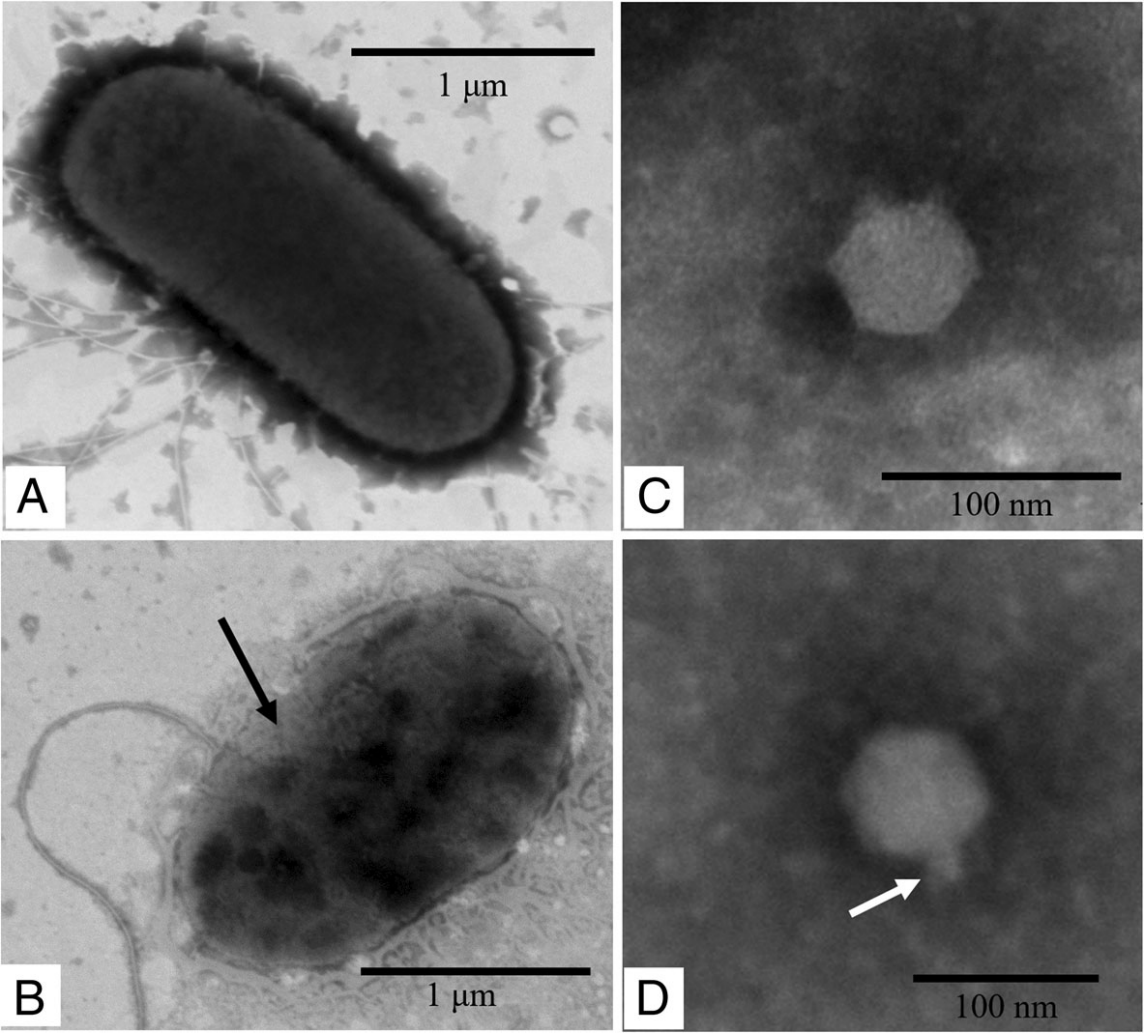
Electron micrograph of *V. alginolyticus* and phage Vp670. **a** A normal *V. alginolyticus* cell. **b** An infected *V. alginolyticus* cell on the verge of lysis. Numerous phage particles (shown by a black arrow) have been assembled within the cell. **c** The morphology of phage Vp670. **d** The short tail of phage Vp670 is shown by the black arrow

The diameter of the plaques produced by phage Vp670 was approximately 4–5 mm after overnight incubation (Additional file 4: Figure S1). Observation by TEM showed that, compared to normal *V. alginolyticus* E06333 cells (Fig. 1a), the cell walls of the infected cells was destroyed, and the outer membrane (OM) layer of the infected cells vanished (Fig. 1b). The infected host cells were filled with phage particles (Fig. 1b). Phage Vp670 has an icosahedral head with a diameter of approximately 55 nm and a short tail approximately 15 nm in length (Fig. 1c and d), which indicated that Vp670 may be a member of the family Podoviridae.

### The burst size, latent period, and phage adsorption rate

**Fig. 2:**
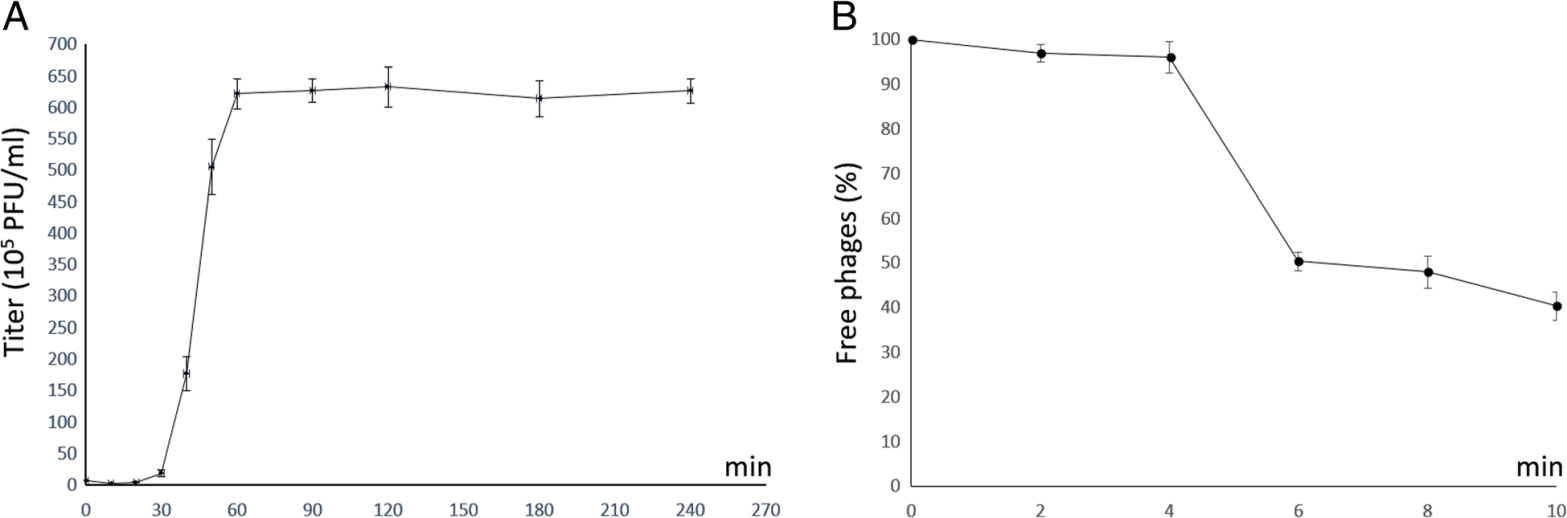
One-step growth curve and adsorption rate of phage Vp670. **a** One-step growth curve of phage Vp670. **b** Adsorption rate of phage Vp670. Triplicate samples were used in each assay

Figure 2a shows the one-step growth curve of phage Vp670. The latent period lasts for the first 30 min, in which the increase of the titer of phage Vp670 was very slow. Then, there was a rapid increase of plaque forming units (PFU), lasting for 30 min and a plateau phase started at 60 min after the infection. The burst size of Vp670 is approximately 84 PFU/infected cell at an MOI of 1 at 30 °C, in which the phages were allowed to propagate in a one-step infection under the assumption that every bacterial cell was infected by one phage. In 10 min of tested, the phages adsorbed to host cells accounted for 3% of total phages 2 min after the starting point, and the phage adsorption rate reached 50% in another 4 min (Fig. 2b).

### Genome and comparative genomics of phage Vp670

**Fig. 3:**
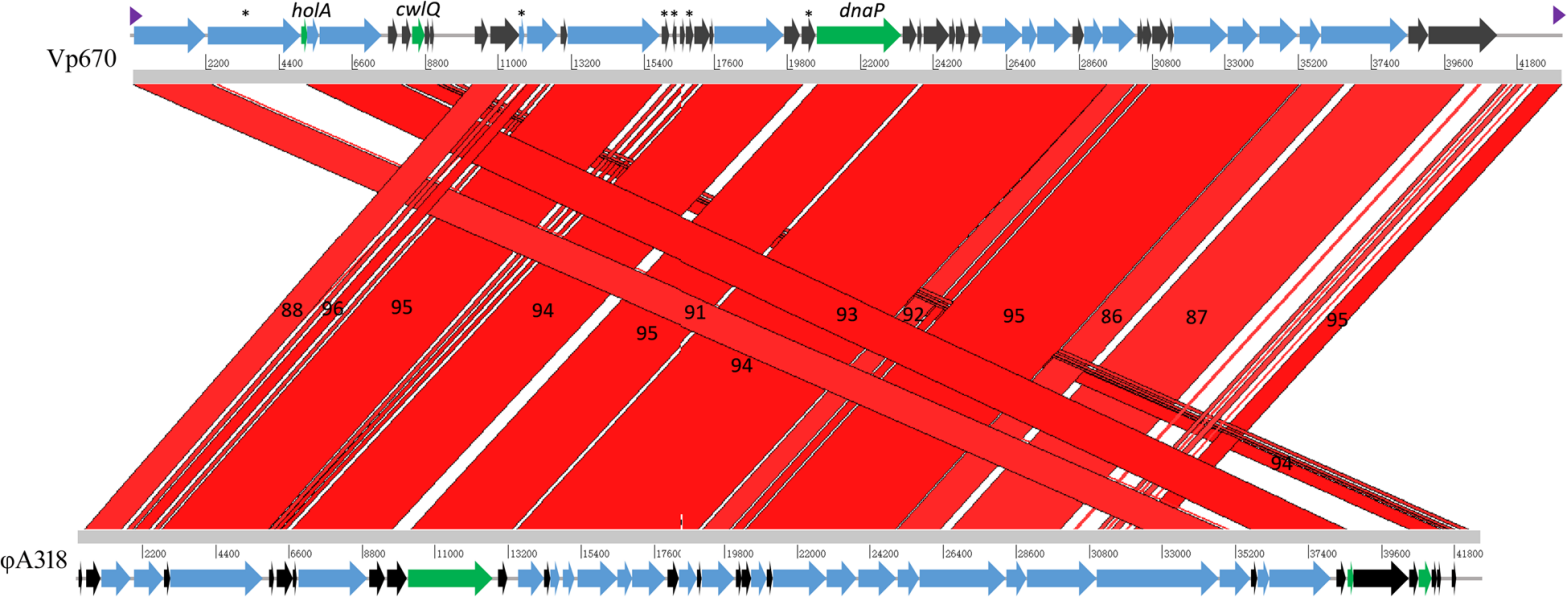
Comparative genomic analysis of phage Vp670 and closely related phage φA318. Comparisons between regions < 50 bp are filtered. Red areas indicate homologous regions and the numbers in them show the identity values of the compared regions. Blue arrows represent the ORFs coding for known proteins. Black arrows represent the ORFs coding for hypothetical proteins. Green arrows represent the interesting genes analyzed in this study. Asterisks represent unique ORFs found in the genome of Vp670. Purple triangular symbols at the ends of Vp670 genome represent two direct repeats

Using Miseq sequencing, we obtained the complete genomic sequence of bacteriophage Vp670, which contains 43,121 nucleotides with a GC content of 43.4%. The Vp670 sequence was deposited in GenBank under the accession number KY290756. There are 49 predicted Open Reading Frames (ORFs) in the Vp670 genome (Table 1). No tRNA-encoding genes and no toxin genes were identified. Among 49 predicted ORFs, 24 ORFs were found to be similar to functionally characterized genes in the database whereas 25 ORFs encode for hypothetical proteins with unknown functions. Only one ORF, *orf18*, was located on the negative strand, and the other 48 ORFs were located on the positive strand (Fig. 3). This arrangement is identical to the arrangement of the genes of the classical T7 phage and BPP-1-like phage in the Podoviridae family [22]. The backbone of the Vp670 genome is divided into four modules, and each module consisted of several functionally related genes. These modules include the replication and regulation module (*orf13*, *orf14*, *orf16*, *orf23*, *orf26*, *orf34*, *orf35*, *orf37*, and *orf38*), the lysis module (*orf3* and *orf8*), the packaging module (*orf4* and *orf5*), and the structure module (*orf43*, *orf44*, *orf45*, *orf46*, *orf47*, *orf1*, and *orf2*).

**Table 1:** Predicted ORFs of *Vibrio alginolyticus* phage Vp670^a^

ORFs	Start	End	Length (bp)	Molecular mass (kD)	Coding for
1	114	2267	2153	78.94	Virion protein
2	2385	5204	2819	103.36	Phage T7 fiber protein
3	5213	5398	185	6.78	Phage holin, HolA
4	5400	5744	344	12.61	Small terminase subunit
5	5744	7618	1874	68.71	Large terminase subunit
8	8496	8885	389	14.26	Endolysin, CwlQ
13	11,660	11,821	161	5.90	Caskin-2/caskin-1
14	11,894	12,826	932	34.17	Protein kinase
16	13,113	15,899	2786	102.15	DNA-dependent RNA polymerase
23	17,580	19,676	2096	76.85	Hydrolase/Topoisomerase-primase
26	20,690	23,233	2543	93.24	Putative DNA polymerase
33	25,816	27,036	1220	44.73	Phage exonuclease
34	27,020	27,457	437	16.02	Endonuclease VII
35	27,457	28,473	1016	37.25	Calcineurin-like Phosphoesterase
37	28,865	29,410	545	19.98	Phosphomevalonate kinase
38	29,410	30,420	1010	37.03	Adenylation DNA ligase-like protein
41	30,888	31,352	464	17.01	Putative acetyltransferase
43	31,537	33,165	1628	59.69	Phage head to tail connecting protein
44	33,165	34,064	899	32.96	Scaffolding protein
45	34,112	35,263	1151	42.20	Major capsid protein
46	35,331	35,978	647	23.72	Tail protein
47	35,981	38,602	2621	96.10	Tail protein
48	38,612	39,217	605	22.18	Putative internal virion protein A

^a^A total of 26 ORFs are predicted to encode for hypothetical proteins, which are omitted from the table

**Fig. 4:**
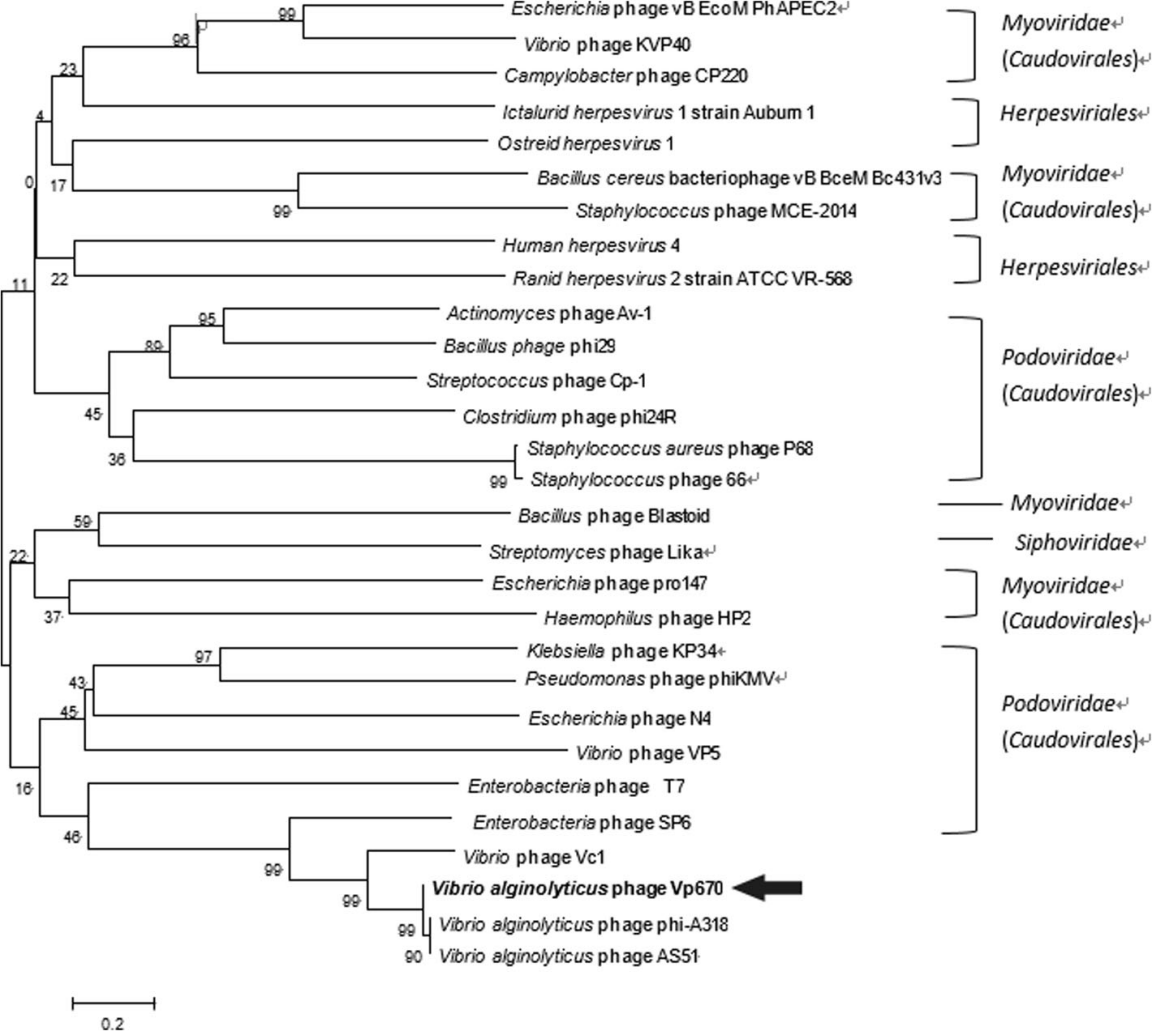
Phylogenetic tree based on the DNA polymerase sequences of phages in the family of Podoviridae*.* The tree was constructed using the neighbor-joining method. It shows that the position of *V. alginolyticus* phage Vp670 (shown by the black arrow) is within the subfamily Autographivirinae, closely related to Enterobacteria phage SP6. The numbers at the nodes indicate the levels of bootstrap support based on data for 1000 replicates. The bar shows 20% sequence divergence

The whole genomic sequence of Vp670 has 88% similarity to that of bacteriophage φA318 (GenBank: KF322026.1). This similarity was mainly due to two aspects: the genomic collinearity (Fig. 3) and DNAP (Fig. 4), which is generally used as a taxonomic marker [23].

The phylogenetic tree based on DNAP sequences revealed that the subfamily Autographivirinae from that Podoviridae family was grouped into a clade, including Enterobacteria phage T7, Enterobacteria phage SP6, *Klebsiella* phage KP34, and *Pseudomonas* phage phiKMV, separated from the members of Picovirinae (Fig. 4). Moreover, *V. alginolyticus* phage Vp670, *Vibrio* phage Vc1 and two other *V. alginolyticus* phages (φA318 and AS51) are also included in a smaller clade. The DNAP sequences of this clade have 99% similarity with that of Enterobacteria phage SP6, a member of Autographivirinae. In this way, bacteriophage Vp670 was classified as a member of subfamily Autographivirinae.

Despite the 88% sequence similarity between VP670 and φA318, the genomic content and structure of Vp670 were different from those of φA318. The genome of Vp670 was divided into four modules: the replication and regulation module, the lysis module, the packaging module and the structure module. The genes related to regulatory functions are more diverse than those of phage φA318. The Vp670 genome also contains six unique genes that are not found in the φA318 genome (Fig. 3). There are three obvious genomic rearrangements in Vp670 (nt 1–2395, nt 5212–8090, and nt 8225–9149) compared with φA318 (Fig. 3). Three rearrangement regions mainly contain four ORFs that putatively code for a virion protein, a holin, a terminus, and an endolysin protein. Interestingly, although both Vp670 and φA318 are *V. alginolyticus*-infecting phages with high sequence similarity, the Vp670 genome is flanked by a 363-bp terminal redundancy whereas φA318 lacks an obvious terminal redundancy region (Fig. 3). However, we could not rule out that the lack of an obvious φA318 terminal redundancy region was caused by the method of sequencing or assembly. Terminal redundancy is found extensively in phage genomes (e.g., T7, T4, and P1), and it could mean that phages with this feature may adopt the same strategy to replicate genomic DNA.

### Lysis-related genes of Vp670

**Fig. 5:**
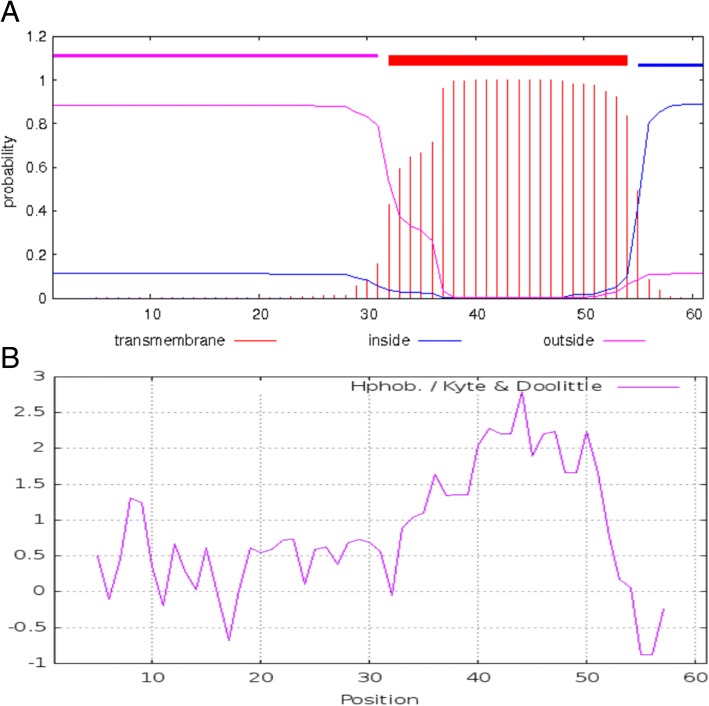
Structural domain and hydrophilicity prediction of HolA from Vp670. **a** Transmembrane domain analysis by TMHMM. **b** Hydrophilicity analysis shows that HolA has a hydrophilic C terminus

In phage Vp670, *orf8* has 99% identity with a putative hydrolase gene from φA318. BLASTX also showed that the hypothetical product of *orf8* has low identity values (37–39%) with a series of cell wall hydrolases from a wide variety of sources. Further searches using SMART showed that the predicted protein encoded by *orf8* has a hydrolase-2 superfamily domain (Pfam 07486). No other ORFs were found to code for an endolysin. Therefore, we speculated that *orf8* (designated as *cwlQ*) represents a novel endolysin, named CwlQ. *orf3* (designated *holA*) is located upstream of *orf8* and encodes a putative phage holin, HolA. HolA was predicted to contain a transmembrane helix (Fig. 5a) with a hydrophilic C-terminal region inside the cytoplasmic membrane (Fig. 5b).

### The effect of the expression of *holA* and *cwlQ* on *E. coli* and V. alginolyticus cells

**Fig. 6:**
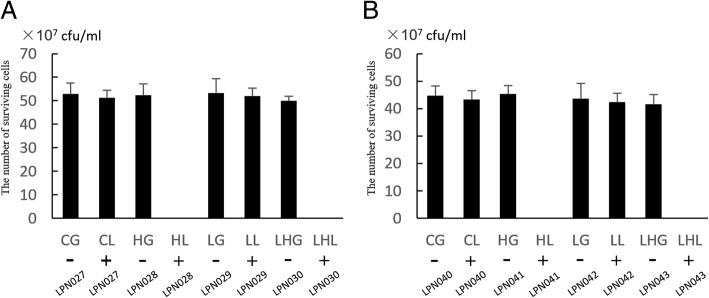
The impact of the expression of *holA* and *cwlQ* on the survival of the host cells. “-” represents the blocked expression of target genes by D-glucose and “+” represents the expression of target genes activated by L-arabinose. **a** Quantification of the survival of *E. coli* cells carrying different recombinant plasmids. LPN027 (pBAD18kan), LPN028 (pBAD18-*holA*), LPN029 (pBAD18-*cwlQ*), and LPN030 (pBAD18-*holA*-*cwlQ*). **b** Quantification of the *V. alginolyticus* cells carrying different expression plasmids. LPN040 (pBAD18kan), LPN041 (pBAD18-*holA*), LPN042 (pBAD18-*cwlQ*), and LPN043 (pBAD18-*holA*-*cwlQ*). Triplicate samples were used in each assay

To explore the function of *holA* and *cwlQ*, we calculated the number of colonies on LB plates supplemented with D-glucose or L-arabinose. The results indicated that the *E. coli* (LPN028 and LPN030) and *V. alginolyticus* (LPN041 and LPN043) cells expressing HolA or HolA and CwlQ could not survive under the L-arabinose induction conditions (Fig. 6a and b). This demonstrated that not only the coexpression of *holA* and *cwlQ* kills all the recombinant cells but also the expression of *holA* alone can kill the induced cells without the participation of induced CwlQ. The expression of endolysin gene alone, c*wlQ*, has no obvious negative effect on *E. coli* LPN029 (Fig. 6a) or *V. alginolyticus* LPN042 (Fig. 6b).

**Fig. 7:**
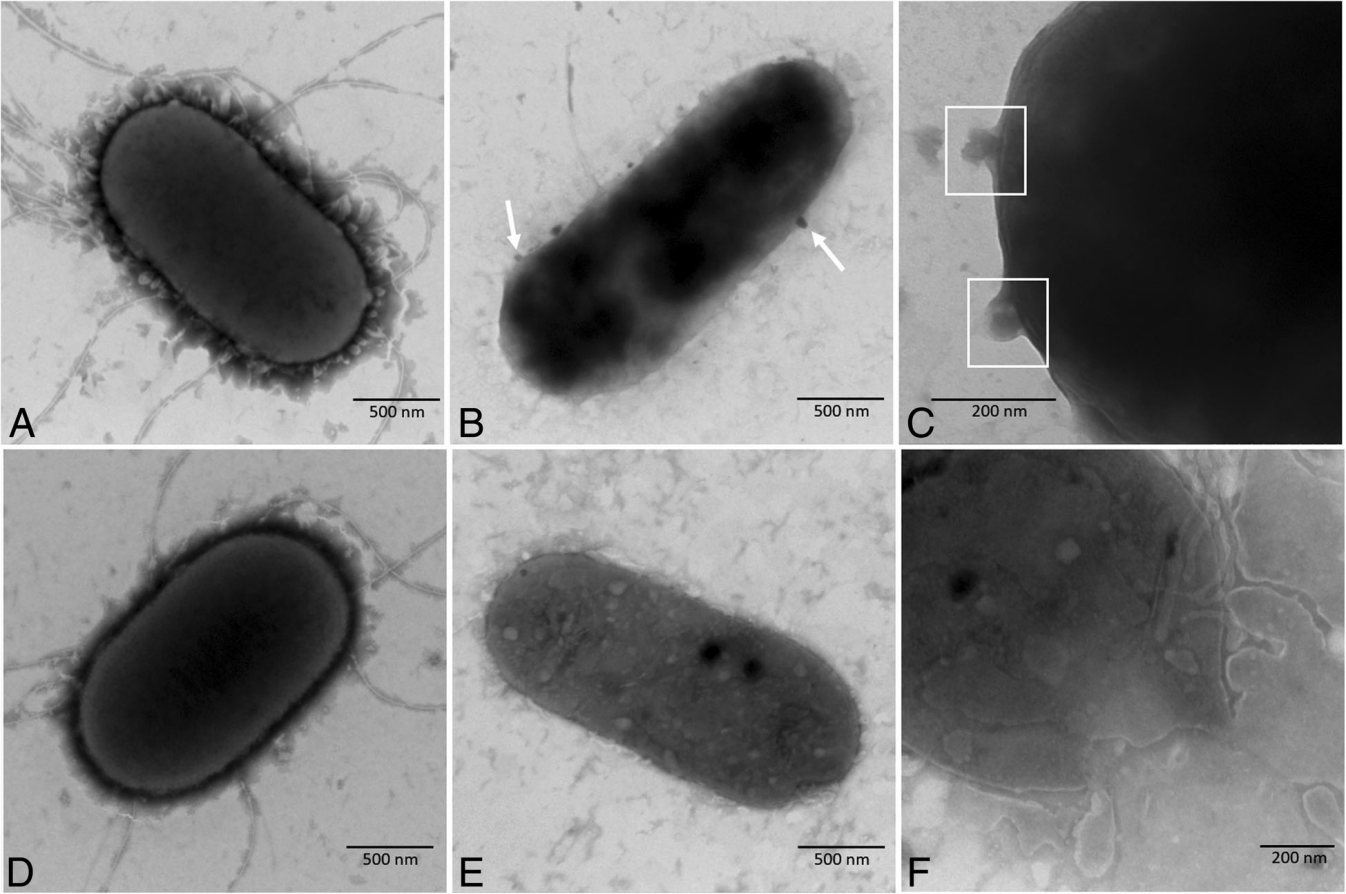
Electron micrograph of the induced *V. alginolyticus* cells. **a** LPN040 cells without the expression of *holA* or *cwlQ*. **b** LPN041 cells with the expression of *holA*. White arrows show the bubbles on the surfaces of the cells. **c** Enlarged image of a LPN041 cell that expresses *holA*. White boxes indicates two bubbles. **d** LPN042 cells with the expression of *cwlQ*. **e** LPN043 cells with the expression of *holA* and *cwlQ*. **f** Enlarged image of a LPN043 cell that expresses *holA* and *cwlQ*

To further verify the functions of *holA* and *cwlQ*, the induced *V. alginolyticus* cells LPN040, LPN041, LPN042, and LPN043 were also observed using TEM. LPN040 cells without *holA* and *cwlQ* expression and LPN042 cells with *cwlQ* expression had intact cellular structures (Fig. 7a and Fig. 7d), and there was no obvious morphological difference between them. The OM layer of induced LPN041 cells expressing HolA either disrupted or vanished and their cellular contents were released from some channels in the cell membrane (Fig. 7b), forming some bubbles outside the cell membrane (Fig. 7c). The release of cellular contents resulted in some intracellular light-colored regions (Fig. 7b). However, the coexpression of *holA* and *cwlQ* in LPN043 cells caused more serious damage to the structure of the cells than the expression of *holA* alone in the cells. Staining of whole LPN043 cells with induced HolA and CwlQ revealed that they had become shallow and rough textured. Many particles were also observed inside the cells (Fig. 7e), which represented a larger loss and condensation of cellular contents. Drastic destruction by induced HolA and CwlQ was also confirmed by the observation of a large breach that allowed the release of numerous cellular contents (Fig. 7f). These results verified that the expression of *holA* destroyed the cellular membrane and killed the induced cells and that coexpression of *holA* and *cwlQ* could induce cell bursting and cause more drastic release of cellular contents.

## Discussion

*V. alginolyticus* is a ubiquitous bacterium found in the marine environment. It has been associated with disease in aquatic animals but also in humans, causing tissue damage in the skin, ears and internal organs [24–26]. Like phage therapy against other pathogenic *Vibrio* species such as *V. parahaemolyticus* [27] and *V. vulnificus* [28], phage therapy also represents a promising strategy for biocontrol of the infection by *V. alginolyticus* [13]. However, until now, few *V. alginolyticus* phages were isolated and subjected to functional genomic analysis. In this study, *V. alginolyticus* phage Vp670 was isolated and its taxonomy, genomic contents, infection kinemics, and the functions of lysis-related genes were characterized.

Phylogenetic analysis based on DNAP sequences indicated that Vp670 is closely related to two *V. alginolyticus* phages, φA318 and AS51, and belongs to Podoviridae family. Compared with the burst sizes of φA318 (72 PFU/cell) [17] and another *V. alginolyticus* phage φGrn1 (44 PFU/cell) [14], Vp670 had a relatively higher burst size (approximately 84 PFU/infected cell), which may imply that Vp670 has a strong *V. alginolyticus* infection ability.

Phages in Podoviridae family are characterized by an isomorphous icosahedral head and a short noncontractile tail [17]. Most tailed phages cause correctly timed host cell lysis through the consecutive action of a two-component lysis system composed of endolysin and holin [29, 30]. In present study, *orf3* (*holA*) was predicted to encode a phage holin, HolA, by our BLAST search. The putative HolA contains a transmembrane helix with a hydrophilic C-terminal region inside the cytoplasmic membrane. These features of HolA are similar to the description of common holing characteristics [31], which further strengthened our speculation that *holA* encodes for a holin. Holins are produced during the late stages of infection, and once a critical concentration is reached, holins form holes in the cytoplasmic membrane by oligomerization, allowing endolysins, which have accumulated in the cytoplasm, to access their PG substrate [32, 33]. Holins trigger the formation of holes that collapse the membrane proton motive force, leading to immediate growth cessation and cell death [30, 34]. In this study, we also observed that not only coexpression of *holA* and *cwlQ* killed all the recombinant cells but also of expression of *holA* alone could completely arrest the growth of *E. coli* and *V. alginolyticus* cells. Further TEM observation confirmed that HolA killed the induced *V. alginolyticus* cells through destroying cellular structures and causing the release of cellular contents. Typical holins spontaneously assemble into oligomers, resulting in the formation of nonspecific and very large pores in the cytoplasmic membrane that allow the nonspecific release of endolysins and other proteins [31, 35, 36]. Though the expression of *holA* had ability to kill and disrupt the integrity of the bacterial cell wall, it could not rapidly cause large breaches on the cell walls and burst the cells without the synergistic effects of the *cwlQ* expression. Apparently, big breaches or cellular burst facilitate the release of phage progeny particles.

Interestingly, though *holA* originates from *V. alginolyticus* phage Vp670, the heterogenic expression of *holA* killed *E. coli* cells as well. This is not the only such case, and similar phenomena have been observed. Heterogenic expression of holin genes from various sources exhibits toxicity towards *E. coli* cells [31, 37, 38]. It suggests that the integration of holins into cell membranes may lack species specificity [31, 38], and therefore, *holA* and even other holin genes are potentially valuable for some applications. For instance, *holA* may be used as a counterselective marker for constructing suicide vectors instead of the unsatisfactory *sacB* gene, which is not universal enough for use in the manipulation of bacterial genetics [21]. As holins can form nonspecific tunnels in bacterial cytomembranes [39], holin-based lytic systems have the potential to function as deliver systems, introducing drugs, nucleic acids, and proteins into eukaryotic cells [40].

The predicted CwlQ exhibits typical features of endolysins from phages of gram-negative bacteria: small molecular mass (between 15 and 20 kDa) and the lack of specific cell wall binding domains [41, 42]. In this study, coexpression of *cwlQ* and *holA* in *V. alginolyticus* thoroughly destroyed bacterial cell walls and burst the cells, causing more drastic damage than the expression of *holA* alone. This implied that *cwlQ*-encoded CwlQ acts as an endolysin but further verification of its function needs to be performed.

## Conclusions

Phage Vp670 is a *V. alginolyticus* phage belonging to Podoviridae family. Phage Vp670 could rapidly absorb to the surface of *V. alginolyticus* cells and lysed the host cells with a burst size of approximately 84 PFU/infected cell at an MOI of 1. The Vp670 genome has a moderate size of 43,121 nucleotides coding for 49 ORFs and contains a two-component lysis module, which is composed of *holA* and *cwlQ*. *holA* is predicted to encode for the holin protein, HolA, and *cwlQ* is predicted to encode for the endolysin protein, CwlQ. The expression of *holA* inside *E. coli* and *V. alginolyticus* cells exhibited strong toxicity to them, but the expression of *cwlQ* alone inside *V. alginolyticus* cells did not generate obvious negative effects on the growth and structure of the cells. Both *holA* and *cwlQ* likely play important roles during the release of progeny phages.

## Additional files

Additional file 1: **Table S1.** The bacterial strains and plasmids used in this study. (DOCX 24 kb)

Additional file 2: **Table S2.** The accession numbers of DNA sequences of phages used for phylogenetic analysis in this study. (DOCX 30 kb)

Additional file 3: **Table S3.** The primers used in this study. (DOCX 24 kb)

Additional file 4: **Figure S1.** Phage plaques of Vp670 infecting *V. alginolyticus* E06333. (TIF 92 kb)

## Abbreviations

DNAP: DNA polymerase
LB: Luria-Bertani
MOI: Multiplicity of Infection
OD: optical density
OM: outer membrane
ORF: Open Reading Frame
PFU: plaque forming units
PG: peptidoglycan
TEM: transmission electron microscopy
