# Supplementary material for: Complete genomic sequence of the Vibrio alginolyticus bacteriophage Vp670 and characterization of the lysis-related genes, cwlQ and holA
Source: BMC Genomics. 2018 Oct 11;19:741. doi: 10.1186/s12864-018-5131-x (PMC6180450; doi:10.1186/s12864-018-5131-x)
Supplement: Supplementary file 1 — Table S1. The bacterial strains and plasmids used in this study. (DOCX 24 kb) [file 12864_2018_5131_MOESM1_ESM.docx]

**Table S1** Bacterial strains used in this study

| **Strain or plasmid** | **Description** | **Reference or source** |
| --- | --- | --- |
| *V. alginolyticus* |  |  |
| E06333 | Isolated from the ulcers of *Epinephelus daemelii* | This study |
| LPN041 | E06333 pBAD18-holA (Kn^R^) | This study |
| LPN042 | E06333 pBAD18-clwQ (Kn^R^) | This study |
| LPN043 | E06333 pBAD18-holA-clwQ (Kn^R^) | This study |
| *E. coli* |  |  |
| DH5α | F- endA1 glnV44 thi-1 recA1 relA1 gyrA96 deoR unpG purB20φ80dlacZΔM15 Δ(lacZYA-argF)U169 hsdR17(r_K_^–^ m_K_^+^) λ^–^ | TaKaRa |
| LPN028 | DH5α pBAD18-holA (Kn^R^) | This study |
| LPN029 | DH5α pBAD18-clwQ (Kn^R^) | This study |
| LPN030 | DH5α pBAD18-holA-clwQ (Kn^R^) |  |
