# Supplementary material for: Complete genomic sequence of the Vibrio alginolyticus bacteriophage Vp670 and characterization of the lysis-related genes, cwlQ and holA
Source: BMC Genomics. 2018 Oct 11;19:741. doi: 10.1186/s12864-018-5131-x (PMC6180450; doi:10.1186/s12864-018-5131-x)
Supplement: Supplementary file 2 — Table S2. The accession numbers of DNA sequences of phages used for phylogenetic analysis in this study. (DOCX 30 kb) [file 12864_2018_5131_MOESM2_ESM.docx]

**Table S1** The accession numbers of DNA sequences of phages used in this study

| Phage name | Accession number |
| --- | --- |
| *Escherichia coli* phage vB EcoM PhAPEC2 | NC_024794.1 |
| *Vibrio* phage KVP40 | NC_005083.2 |
| *Campylobacter* phage CP220 | NC_027997.1 |
| *Lctalurid herpesvirus* 1 strain Auburn 1 | NC_001493.2 |
| *Ostreid herpesvirus* 1 | NC_005881.2 |
| *Bacillus cereus* bacteriophage vB BceM Bc431v3 | NC_020873.1 |
| *Staphylococcus* phage MCE-2014 | NC_025416.1 |
| *Human herpesvirus* 4 | NC_007605.1 |
| *Ranid herpesvirus* 2 strain ATCC VR-568 | NC_008210.1 |
| *Actinomyces* phage Av-1 | NC_009643.1 |
| *Bacillus* phage phi29 | NC_011048.1 |
| *Streptococcus* phage Cp-1 | NC_001825.1 |
| *Clostridium* phage phi24R | NC_019523.1 |
| *Staphylococcus aureus* phage P68 | NC_004679.1 |
| *Staphylococcus* phage 66 | NC_007046.1 |
| *Bacillus* phage Blastoid | NC_022773.1 |
| *Streptomyces* phage Lika | NC_021298.1 |
| *Escherichia* phage pro147 | NC_028896.1 |
| *Haemophilus* phage HP2 | NC_003315.1 |
| *Klebsiella* phage KP34 | NC_013649.2 |
| *Pseudomonas* phage phiKMV | NC_005045.1 |
| *Escherichia* phage N4 | NC_008720.1 |
| *Vibrio* phage VP5 | NC_005891.1 |
| *Enterobacteria* phage T7 | NC_001604.1 |
| *Enterobacteria* phage SP6 | NC_004831.2 |
| *Vibrio* phage Vc1 | KJ502657.1 |
| *Vibrio alginolyticus* phage phi-A318 | NC_025822.1 |
| *Vibrio alginolyticus* phage AS51 | KF800937.1 |
| *Vibrio alginolyticus* phage Vp670 | KY290756 |
