# Supplementary material for: Complete genomic sequence of the Vibrio alginolyticus bacteriophage Vp670 and characterization of the lysis-related genes, cwlQ and holA
Source: BMC Genomics. 2018 Oct 11;19:741. doi: 10.1186/s12864-018-5131-x (PMC6180450; doi:10.1186/s12864-018-5131-x)
Supplement: Supplementary file 3 — Table S3. The primers used in this study. (DOCX 24 kb) [file 12864_2018_5131_MOESM3_ESM.docx]

**Table S1** The DNA sequences of the primers used in this study

| Primer name | Nucleotide sequence (5′ to 3′)*^a^* | Use in this study |
| --- | --- | --- |
| pBAD18-recF | CTGCAGGCATGCAAGCTTGG | Linearize the vector for recombination *in vitro* |
| pBAD18-recR | GGTACCGAGCTCGAATTCGC |  |
| pBAD18-TF | GATTAGCGGATCCTACCTGACGC | Test correct recombinated plasmids |
| pBAD18-TR | CTGATTTAATCTGTATCAGGCTG |  |
| holA-exF | GCGAATTCGAGCTCGGTACCAGGAGGAATTCACCGTGAGTGAAAAGATTGTAAAAGC | Amplify holA gene with an adapter |
| holA-exR | CCAAGCTTGCATGCCTGCAGTTAAATCCCCTTTCTCCACTTAT |  |
| cwlQ-exF | GCGAATTCGAGCTCGGTACCAGGAGGAATTCACCATGTTCGTTGAAGCAATTCTATG | Amplify cwlQ gene with an adapter |
| cwlQ-exR | CCAAGCTTGCATGCCTGCAGTCAAGGTCTTCCTCCATTATC |  |
| holA-clwQ-exF1 | the same sequence with that of holA-exF | Amplify fused holA and cwlQ gene through overlap PCR |
| holA-clwQ-exR1 | ATTGCTTCAACGAACATATTAAATCCCCTTTCTCCAC |  |
| holA-clwQ-exF2 | GTGGAGAAAGGGGATTTAATATGTTCGTTGAAGCAAT |  |
| holA-clwQ-exR2 | the same sequence with that of cwlQ-exR |  |

*^a^* Restriction sites are underlined
