# Supplementary figures and images for: Complete genomic sequence of the Vibrio alginolyticus bacteriophage Vp670 and characterization of the lysis-related genes, cwlQ and holA
Source: BMC Genomics. 2018 Oct 11;19:741. doi: 10.1186/s12864-018-5131-x (PMC6180450; doi:10.1186/s12864-018-5131-x)

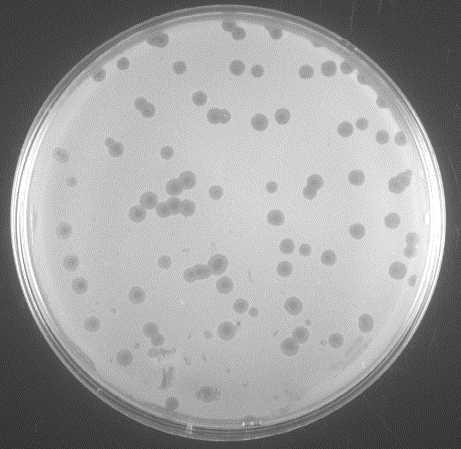

Supplement: Supplementary file 4 — Figure S1. Phage plaques of Vp670 infecting V. alginolyticus E06333. (TIF 92 kb) [file 12864_2018_5131_MOESM4_ESM.tif]
